# Recent Advances on Electrospun Nanofibers for Periodontal Regeneration

**DOI:** 10.3390/nano13081307

**Published:** 2023-04-07

**Authors:** Mafalda S. Santos, Marta S. Carvalho, João C. Silva

**Affiliations:** 1 Department of Bioengineering, iBB-Institute for Bioengineering and Biosciences, Instituto Superior Técnico, Universidade de Lisboa, Av. Rovisco Pais, 1049-001 Lisboa, Portugal; mafaldasantos4@tecnico.ulisboa.pt; 2 Associate Laboratory i4HB-Institute for Health and Bioeconomy, Instituto Superior Técnico, Universidade de Lisboa, Av. Rovisco Pais, 1049-001 Lisboa, Portugal

**Keywords:** periodontium, periodontitis, nanofibers, biocompatible materials, periodontal regeneration, tissue engineering

## Abstract

Periodontitis is an inflammatory infection caused by bacterial plaque accumulation that affects the periodontal tissues. Current treatments lack bioactive signals to induce tissue repair and coordinated regeneration of the periodontium, thus alternative strategies are needed to improve clinical outcomes. Electrospun nanofibers present high porosity and surface area and are able to mimic the natural extracellular matrix, which modulates cell attachment, migration, proliferation, and differentiation. Recently, several electrospun nanofibrous membranes have been fabricated with antibacterial, anti-inflammatory, and osteogenic properties, showing promising results for periodontal regeneration. Thus, this review aims to provide an overview of the current state of the art of these nanofibrous scaffolds in periodontal regeneration strategies. First, we describe the periodontal tissues and periodontitis, as well as the currently available treatments. Next, periodontal tissue engineering (TE) strategies, as promising alternatives to the current treatments, are addressed. Electrospinning is briefly explained, the characteristics of electrospun nanofibrous scaffolds are highlighted, and a detailed overview of electrospun nanofibers applied to periodontal TE is provided. Finally, current limitations and possible future developments of electrospun nanofibrous scaffolds for periodontitis treatment are also discussed.

## 1. Introduction

Periodontitis is a chronic inflammatory infection of the periodontium, the structure responsible for ensuring tooth attachment and stability [1]. Bacterial dental plaque accumulation causes this infection, which can lead to the inflammation and destruction of the periodontium and, ultimately, tooth loss [2]. Advanced stages of the disease require regenerative procedures to restore the lost tissues, which include membranes for guided tissue regeneration (GTR) and bone grafts. However, these current treatments lack effective strategies to induce tissue repair and coordinated regeneration of all periodontal tissues, increasing the demand for alternative solutions to improve clinical outcomes [3].

Nanofibrous scaffolds have been widely explored in tissue engineering (TE) strategies due to their unique properties such as high porosity, surface area, and interconnectivity. Electrospinning allows the efficient production of nanofibrous scaffolds that can mimic the morphology and scale of native extracellular matrix (ECM) proteins, thus promoting cell attachment, proliferation, and differentiation [4]. Through the optimization of electrospinning parameters and selection of the most adequate polymers and additives, it is possible to tailor the characteristics of electrospun polymeric nanofibrous scaffolds for a desired purpose. Nanofibrous scaffolds with high porosity and small pore size are highly suitable for the development of novel periodontal barrier membranes [5]. Specific additives incorporated in scaffolds, such as antibiotics and bioceramics, can provide antibacterial activity against oral pathogens and osteogenic properties to promote tissue regeneration, which are advantageous features for periodontitis treatment [6]. Furthermore, the biomimetic characteristics of nanofibers can be exploited for the development of more suitable constructs to insert in periodontal defects as alternatives to bone grafts.

Recently, substantial research has been carried out regarding the development of electrospun nanofibrous scaffolds for periodontal regeneration. Nanofibers produced from a wide variety of polymers and composites, with diverse biochemical factors incorporated, have been studied and have shown promising results in in vitro and in vivo settings. This review covers the principal features of periodontal tissues, describes the main causes and symptoms of periodontal disease, and summarizes the currently available treatments. Alternative strategies proposed by periodontal TE are explored. The electrospinning technique is detailed and the features of electrospun nanofibrous scaffolds are outlined. The recent research on electrospun nanofibrous scaffolds for periodontal regeneration is summarized and discussed. Finally, shortcomings and future perspectives of using electrospun nanofibrous scaffolds for periodontitis treatment are debated.

## 2. Periodontal Tissue Features and Periodontal Disease

**Figure 1:**
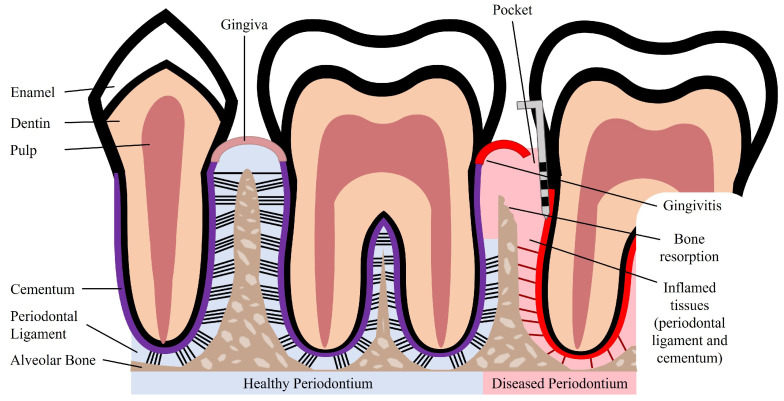
Schematic illustration of a healthy and diseased periodontium.

The periodontium is a complex structure composed of hard and soft tissues that support the tooth. It has an important role of ensuring tooth attachment to the bone of the jaw and allowing the teeth to withstand the forces of mastication. The periodontium consists of alveolar bone, root cementum, and periodontal ligament (PDL) [7]. The anatomy of the periodontium is illustrated in Figure 1.

The alveolar bone is the part of the maxilla or mandible that contains the sockets that surround and anchor the teeth. The alveolar bone is a highly mineralized, hard tissue composed of 60% (*w*/*w*) inorganic material, 25% (*w*/*w*) organic material, and 15% water [8]. In the root of the teeth, the alveolar bone is connected to the root cementum through the PDL, as can be observed in Figure 1. The alveolar bone is perforated by channels, which allow the passage of blood vessels and nerve fibers that extend to within the pulp of the teeth [9]. Similar to what occurs in other types of bones, alveolar bone is maintained through constant bone remodeling. Because the teeth are continuously making minor movements and there is a functional demand due to the forces of mastication, the alveolar bone undergoes constant remodeling. Bone remodeling relies on a balance between bone resorption and bone deposition, which is maintained by progenitor cells that can differentiate into osteoclasts (bone resorption) and osteoblasts (bone deposition) [10,11].

The cementum is a hard, avascular connective tissue that covers the roots of teeth. It is located between the dentin and the PDL, as can be seen in Figure 1. The primary function of the cementum is to anchor the PDL fibers. The cementum’s composition is very similar to that of the alveolar bone, namely 65% (*w*/*w*) inorganic material, 23% (*w*/*w*) organic material, and 12% water [8]. The organic material is constituted by up to 90% of collagen type I. Interestingly, the majority of non-collagenous matrix proteins present in the cementum are also found in bone, namely fibronectin, osteocalcin, osteonectin, and osteopontin [12]. The cementum is produced as repair tissue to fill root fractures and resorptive defects [9,12]. In periodontal regeneration, new cementum is formed from cementoblasts. Reports suggest that the PDL serves as a source of progenitor cells for cementoblasts involved in cementum formation and also for osteoclasts and osteoblasts involved in bone remodeling [10,11].

The PDL is a complex, highly cellular, fibrous connective tissue located between the alveolar bone and the cementum, as can be seen in Figure 1. The width of the PDL ranges between 100 and 400 µm; however, it progressively decreases in thickness with age [10,13]. The extracellular compartment of the PDL is composed of highly aligned and organized collagen fiber bundles and non-collagenous matrix constituents, such as glycoproteins and proteoglycans [14]. The collagen fiber bundles provide the structural strength of the PDL and are mainly composed of collagen type I [8]. The fibers and fibrils present in the PDL are in the scale of nanometers to micrometers [9,13]. The extremities of the collagen fiber bundles are embedded in cementum or alveolar bone. The PDL is primarily responsible for providing support and mechanical stability to the teeth. It connects the cementum covering the tooth to the alveolar bone, ensuring the attachment of the tooth to the bone, while absorbing the shock from the considerable forces associated with mastication [3,14]. When characterized through tensile testing under loads between 1 and 5 N, the PDL demonstrated values of elastic modulus in the range between 0.607 and 4.274 MPa. Its elastic behavior is influenced by the loading rate, type of tooth, root level, and individual variability [15]. Furthermore, the PDL is innervated and can act as a sensory receptor for regulating pressure on the teeth and proper positioning of the jaw during mastication.

The PDL possesses an extensive blood supply and a diversity of cell populations, which include osteoblasts, osteoclasts, cementoblasts, fibroblasts, and progenitor cells [16] Another cell population that is present in the PDL are periodontal ligament stem/stromal cells (PDLSCs), which serve as a source for renewable progenitor cells, which can differentiate into osteoblasts, cementoblasts, and fibroblasts. Due to the presence of these heterogeneous cell populations, the PDL serves as a cell reservoir for tissue homeostasis, repair, and regeneration [3,14]. Blood vessels present in the PDL provide nutrients necessary for the maintenance of the ligament and the hard tissues. The PDL connects the root cementum to the alveolar bone and sustains a balance between formation and maintenance of the hard and soft tissues. The unique structure and composition of the PDL is essential for the physiological functionalities of periodontal tissues [3].

Periodontal disease is characterized by an inflammatory infection of the periodontium. This infection is caused and sustained by bacteria from dental plaque accumulation. In early stages of the disease, there is inflammation only of the gingiva, known as gingivitis, which is reversible with effective oral hygiene [1]. However, if left untreated, gingivitis can progress to periodontitis. Periodontitis in its advanced form is characterized by the loss and destruction of the periodontal tissues, including PDL, root cementum, and alveolar bone, as can be seen in Figure 1 [2,17]. This results in the loss of the tooth attachment to its supporting structures of the periodontium and in the formation of pockets surrounding the tooth. The symptoms of severe periodontitis include pain and discomfort during mastication, drifting and mobility of teeth, and tooth loss [18]. Periodontitis is the main cause of tooth loss, which is a global health problem representing a burden to society and the economy, particularly affecting older people [2]. The economic burden of periodontal disease was estimated to be USD 154.06B in the United States and EUR 158.64B in Europe, in 2018 [19]. Periodontitis is prevalent in adults and elderly populations and can also occur in children and adolescents. The prevalence of periodontal disease, which includes gingivitis and periodontitis, is estimated to range from 20% to 50% worldwide [1,17]. This large range of estimated prevalence is due to the absence of a unique and consensual case definition among different countries and populations [18]. Periodontitis can be characterized by the number of affected teeth, the magnitude of the pocket depth, the loss of tooth attachment capacity, and the loss of alveolar bone. More severe forms of periodontitis are estimated to affect 10% of the population [17].

Although bacterial plaque accumulation is the initiator of gingivitis, the host’s susceptibility to disease progression plays an important role. In patients not susceptible to periodontitis, the primary defense mechanisms are able to control the infection, and the inflammation of the gingiva may persist indefinitely without progressing to periodontitis. On the other hand, the primary defenses of patients susceptible to periodontitis cannot contain the infection of the gingiva, and the infection spreads to the periodontium [17]. The destruction of the periodontal tissues is in fact caused by host-derived mediators and enzymes from inflammatory cells in response to the bacterial infection of the periodontium. The inability to control the infection allows it to further progress into the tooth root, deepening the pockets and resulting in tooth attachment loss and alveolar bone loss [1].

Patient susceptibility is significantly affected by risk factors that increase the probability of periodontitis development. The risk factors can be genetic or environmental. Genetic risk factors that increase patient susceptibility to disease include defects of phagocytosis, which leads to an insufficient response to the bacterial infection, and enhanced enzyme production for a bacterial challenge, resulting in an excessive response with increased tissue damage [1]. Environmental or acquired risk factors include smoking, which is associated with decreased wound healing and reduced bacterial killing. Studies show that smokers are more likely to have severe periodontitis, present increased loss of alveolar bone, and have higher prevalence of tooth loss when compared to non-smokers [1,2]. Poor oral hygiene is another risk factor, as it allows accumulation of dental plaque and is linked to increased severity of periodontitis [2].

Furthermore, there is a link between systemic diseases and periodontitis. Periodontitis poses a risk of systemic complications associated with cardiovascular disease, cancer, lung diseases, and diabetes [20]. In fact, diabetes has a bi-directional relationship with periodontitis. Diabetic patients show higher concentrations of inflammatory mediators compared to non-diabetic individuals. The severity and extent of periodontitis is directly influenced by the metabolic control of diabetic individuals [17,21]. Age is a potential risk factor, as the risk of periodontitis increases with the advancing age, with a higher prevalence of the disease in elderly populations [2]. Poor oral health has been shown to be associated with disability and poor physical function in older populations [22]. In addition to motor disability, intellectual or developmental disability can also have an effect on the state of periodontal health [23]. For example, patients suffering from autism have difficulties in correctly applying oral hygiene rules and undergoing dental visits and therapies, which expose these patients to a greater risk in developing periodontitis [24].

## 3. Currently Available Treatments for Periodontal Disease

Initial stages of periodontitis can be treated with non-surgical procedures such as dental plaque and tartar removal with scaling and root planing [1,18]. The main goal of these treatments is to control and reduce bacterial plaque accumulation. After the clinical removal of the dental plaque, the patient should practice adequate oral hygiene to achieve a good clinical outcome [18]. Non-surgical treatments can be combined with adjunctive therapies, such as local drug delivery, systemic antibiotics, and systemic host response modulation to improve treatment outcomes. Adjunctive drugs include antibiotics and antimicrobials that are directly administered to the periodontal pocket via a gel or fiber delivery system. Examples of systemic antibiotics are amoxicillin and metronidazole, which in combination result in pronounced clinical improvements [18]. Host response modulation can be particularly beneficial for patients susceptible to disease development. Host modulatory therapies influence the destructive components of the host response to reduce periodontal tissue destruction. These therapies include non-steroidal anti-inflammatory drugs: doxycycline that downregulates collagenases in inflamed periodontal tissues; and bisphosphonates, which reduce osteoclast activity and bone resorption [1].

**Figure 2:**
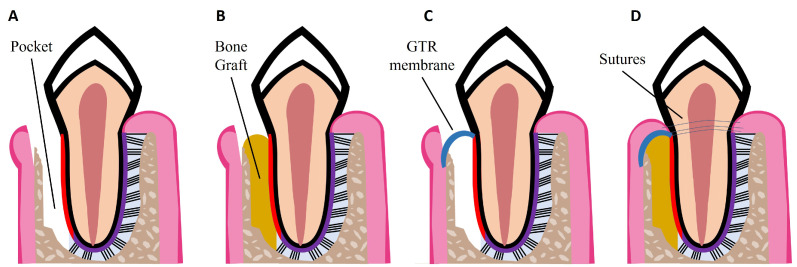
Schematic illustrations of procedures for periodontal regeneration. (**A**) Loss of PDL and alveolar bone, resulting in periodontal pocket formation. (**B**) Bone graft placed in the defect site. (**C**) GTR membrane placed over the defect site. (**D**) Combination of GTR membrane and bone graft. Wound closure with sutures.

Non-surgical treatments have been shown to reduce pocket depth and allow formation of new tooth attachment, which can be sufficient for early to moderate stages of periodontitis. However, in some cases and in advanced stages of the disease, surgical therapy is necessary to access sites deeper in the tooth root to control the inflammation, to fully eliminate bacterial plaque, and to stimulate the regeneration of lost periodontal tissues [18]. Pocket reduction surgery is a procedure that involves resecting soft and hard necrotic tissues. Regenerative surgery includes GTR membranes and bone grafts, which are illustrated in Figure 2. An innovative adjuvant is the use of laser treatment in non-surgical or surgical procedures [25]. The surgical and non-surgical currently available methods used to treat periodontitis are summarized in Table 1.

GTR is based on the use of a mechanical barrier membrane that prevents epithelial cells and fibroblasts from migrating into the defect site while maintaining sufficient space for the regeneration of all the periodontal tissues, namely alveolar bone, cementum, and PDL [34]. There are two types of membranes already commercially available that can be used for periodontal regeneration: non-degradable and degradable membranes. Currently available non-degradable membranes include polytetrafluoroethylene membranes, such as Cytoplast^TM^ TXT-200; however, a second surgery is required for their removal. To avoid additional surgeries, there are degradable membranes on the market, which are composed of synthetic polymers such as polycaprolactone (PCL), polylactic acid (PLA), and polyglycolic acid (PGA), and of natural polymers such as collagen, for example from porcine collagen, which is used in the Bio-Gide^®^ commercially available membrane. However, current GTR membranes have limitations such as low attachment to the adjacent tissues, which can lead to an early exposure of the defect site and allow bacteria infiltration; lack of antibacterial properties; and poor ability to enhance the regeneration of all the periodontal tissues [3,34]. Thus, new improved membranes need to be developed aiming to meet all the criteria for an ideal GTR membrane, namely: biocompatibility; non-immunogenicity as to not trigger adverse reactions; biodegradability without release of toxic byproducts; cell-occlusivity to exclude specific cell types; and ease of use in a clinical setting [35]. They should also possess appropriate surface area and high porosity for cell attachment, proliferation, and differentiation, as well as mechanical strength to stay in place for at least 4–6 weeks and to maintain space for the slow regenerating periodontium. Finally, GTR membranes should present bioactivity to accelerate tissue repair and induce a coordinated regeneration of all the periodontal tissues [35,36]. Considering the slowly regenerating alveolar bone, bone grafts can be used to fill the defect site. GTR membranes can be combined with bone grafts to prevent membrane collapse, as illustrated in Figure 2D.

Bone grafts are transplanted into bone defects, where they promote bone healing either alone or in combination with other materials. Their main functions are to provide mechanical support and enhance bone regeneration [33]. Bone grafts need to have four essential properties for achieving successful bone regeneration: osseointegration, which refers to the graft’s capability to bind to the bone’s surface; osteogenesis, which is the formation of new bone through osteoblasts present in the graft; osteoconductivity, which is the graft’s potential to generate a scaffold on which host cells can grow; and osteoinductivity, which translates to the graft’s ability to recruit host stem cells into it and induce their differentiation into osteoblasts through local proteins and growth factors [37]. Unfortunately, current bone grafts mainly fulfill only the osteoconductivity property by serving as a structure for regeneration processes to occur [38]. Although some bone grafts might present almost all four essential properties for successful bone regeneration, their success is also influenced by the grafts biocompatibility, biodegradability, structural integrity, and porosity [39]. Moreover, the grafts are envisaged for bone formation, thus neglecting PDL regeneration. It is important to note that not only is osseointegration important, but so also is the attachment of newly formed bone to a regenerated PDL, which in turn connects the newly formed bone to the cementum of the tooth. In addition to bone grafts, GTR membranes also fail to achieve PDL regeneration and integration of soft (PDL) and hard tissues (alveolar bone, cementum) [3]. If the PDL is not regenerated, there is no connection between cementum and alveolar bone, and the tooth will eventually be lost due to the lack of attachment to the bone. These regenerative procedures are still exposed to clinical failures and do not effectively promote periodontal regeneration. Therefore, innovative strategies that promote the regeneration of the entire hierarchical structure of the periodontium are needed to improve clinical outcomes.

## 4. Periodontal Tissue Engineering

**Figure 3:**
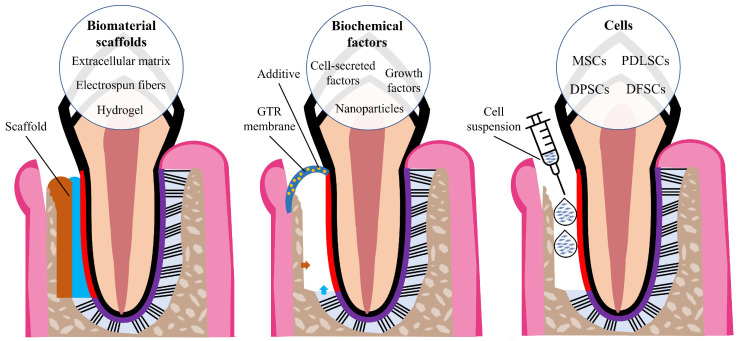
The three key components of TE and examples of periodontal TE strategies (bilayered scaffold, GTR membrane with biochemical additives, and cell suspension).

TE relies on the use or combination of cells, scaffolds, and biochemical factors to facilitate tissue regeneration (Figure 3). TE strategies use or manipulate one or more of these mediators with the aim of promoting the regeneration of diseased or damaged tissues. Various TE strategies for periodontal regeneration have been reported in the literature, proposing alternatives to the current regenerative treatments of bone grafts and GTR membranes used in the treatment of periodontitis.

Mesenchymal stem/stromal cells (MSCs) are commonly used in periodontal TE strategies. MSCs present hypoimmunogenicity and immunomodulatory properties, making them promising candidates for TE applications [40]. MSCs derived from adult tissues (e.g., bone marrow, adipose tissue, and synovial tissue) present no ethical or legal concerns, can be expanded in vitro, and used in TE strategies [41,42]. PDLSCs show similar characteristics to MSCs, such as fibroblast-like morphology, multilineage differentiation capacity, and expression of MSC-related surface markers [43,44,45]. PDLSCs are present in the PDL and serve as a source for renewable progenitor cells, which differentiate into osteoblasts, cementoblasts, and fibroblasts, responsible for bone, cementum, and PDL formation [14]. Stem cells have also been isolated from the dental pulp and the dental follicle. The dental follicle is a loose connective tissue that surrounds the enamel and the dental pulp of the developing tooth germ before tooth eruption, hence dental follicle stem cells (DFSCs) also give rise to progenitors of osteoblasts, cementoblasts, and PDL cells [46].

Current stem cell-based therapies rely mainly on the delivery of cells that were expanded in vitro to the periodontal defect site with the goal of promoting regeneration [47]. This delivery can be performed using single-cell suspensions injected into the defect site, which represents a simple and minimally invasive procedure [48]. Bone marrow-derived MSCs (BMMSCs) have been injected into rat periodontal defect models and showed the capacity to exert anti-inflammatory and immunomodulatory effects and promote periodontal regeneration, as MSCs can differentiate into the osteogenic lineage [47,48,49]. However, injection of single-cell suspensions have drawbacks, including poor engraftment, significant decrease in cell number after implantation, spreading to surrounding tissues, and loss of cell fate control [48]. Because PDLSCs were shown to be highly proliferative and capable of regenerating cementum/PDL-like tissues in vivo [43], interest was raised regarding their potential for use in periodontal tissue regeneration as a stem cell-based therapy to treat periodontal defects. Their regenerative capacity was studied in dental defects using several animal models (e.g., rat, miniature pig, and beagle dog defect models), and results showed that PDLSCs had the potential to form soft and hard periodontal-like structures and to promote periodontal regeneration [43,49,50,51,52]. In addition to single-cell suspensions, another possible stem-cell based approach is the delivery of monolayer or stacked cell sheets. Cell sheets remain intact as a whole due to cellular junctions and ECM. This technique is based on harvesting confluent cultured cells without any enzyme, which is easier to implement than cell suspensions and results in minimized cell loss and higher cell viability [47,48]. Interestingly, a study compared cell injection and cell sheet transplantation of human dental pulp stem cells (DPSCs) in swine periodontal bone defect models. The results showed that both approaches were able to significantly regenerate alveolar bone; however, the cell sheet transplantation exhibited higher bone regeneration capacity [53]. Nevertheless, cell sheets require a longer culture period, are fragile if cells are not confluent enough, attach weakly to hard tissues, and cell sheets that are too thick present necrotic cells. These limitations may be addressed with the use of biomaterial scaffolds. Examples of different sources from which stem cells can be isolated and used for periodontal regeneration are summarized in Table 2.

## 5. Electrospun Nanofibers for Periodontal Tissue Engineering

Tissue-engineered scaffolds made of synthetic polymers, such as PCL, PLA, poly(lactic-co-glycolic acid) (PLGA), and polyethylene glycol (PEG), or of natural polymers, such as chitosan, gelatin, and collagen, have been employed for periodontal regeneration. Synthetic polymers offer tailorable and reproducible structural properties, which allows mass production. Although they present good mechanical properties, they have slow degradation rates and poor biological activity [57]. Natural polymers have high biocompatibility and advantageous bioactivity, associated with enhanced cell adhesion, proliferation, and matrix production [58]. Synthetic and natural polymers can be combined to address their limitations and obtain scaffolds with the advantageous features of both polymer types [59].

Nanofibrous scaffolds possess unique properties, such as high surface area to volume ratio, porosity, and interconnectivity which favor cell attachment and proliferation and also enable nutrient and waste exchange. Electrospinning is a technique for fabricating continuous fibers with an average diameter ranging from few nanometers to micrometers [60]. Cumulative fibers form non-woven fibrous membranes that mimic the morphology of ECM proteins, therefore facilitating cell attachment, proliferation, and differentiation. Electrospinning allows the production of fibrous scaffolds with controllable fiber diameter, fiber orientation, porosity, and surface characteristics [61].

The electrospinning technique is simple, cost-effective, and requires four main components: a syringe containing a polymeric solution, a spinneret with a metallic needle, a high-voltage power supply, and a grounded metal collector. The syringe with the polymeric solution is placed in a pump, which ejects the solution and controls its flow rate. The solution is ejected through the metallic needle that is connected to the high voltage power supply. The power supply is also connected to the metal collector, and an electrostatic field is formed between the needle and the collector. The high voltage makes the droplets formed at the needle tip by electrically charging the polymeric solution. The droplets are stretched with electrostatic forces that counteract the solution’s surface tension into an elongated shape, known as the Taylor cone, from which a jet of charged fluid is pulled towards the grounded collector. The electrospun polymeric fibers deposit and solidify in the collector, and the solvent evaporates during the electrospinning process, resulting in dry fibers on the collector, which accumulate overtime to form fibrous scaffolds [4].

Essentially, electrospinning uses a polymeric solution to fabricate fibers in a high electrostatic field. There are various factors that influence the characteristics of the collected electrospun fibers, and these factors can be divided in three categories: solution parameters, process parameters, and environmental parameters. The solution parameters include solution concentration, solution viscosity, solution surface tension, polymer solubility, and polymer molecular weight. The process parameters consist of the voltage, solution flow rate, needle inner diameter, needle-to-collector distance, and type of collector. The environmental parameters comprise temperature and humidity [62].

**Figure 4:**
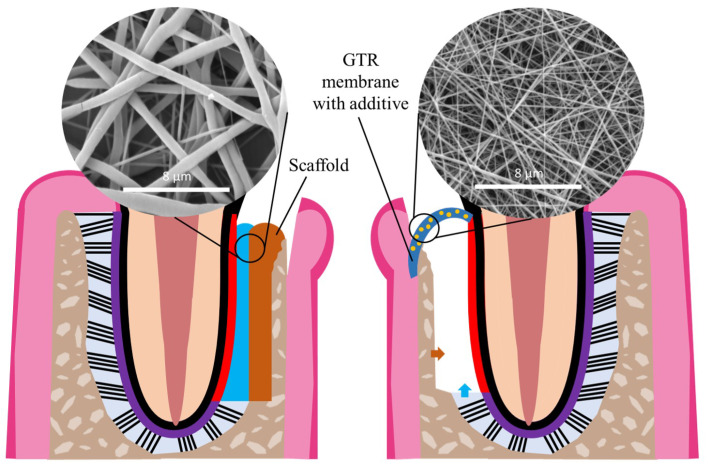
Examples of possible applications of nanofibers in periodontal TE strategies: as scaffold (**left image**) or as GTR membrane (**right image**).

By using the most adequate polymers and through the optimization and consideration of all the parameters that influence the features of electrospun fibers, it is possible to produce electrospun polymeric fibrous scaffolds with the most appropriate properties for a specific application. Electrospinning has been used in various TE strategies, including periodontal TE, as electrospun fibrous scaffolds are highly suitable for the development of periodontal GTR barrier membranes and biomimetic scaffolds, as illustrated in Figure 4. The high porosity with small pore size can prevent the migration of fibroblasts across the nanofibrous scaffolds, which is a vital feature of a GTR membrane [5]. By providing a closer mimicry of the native ECM, electrospun nanofibrous scaffolds can be part of a TE construct that facilitates tissue regeneration as an alternative to bone grafts. Through the use of optimized electrospinning parameters and the combination of carefully selected synthetic and natural polymers, nanofibrous scaffolds can meet the criteria of an ideal GTR membrane: biodegradable, biocompatible, osteoinductive, and having good mechanical properties [36]. The properties of electrospun fibers are summarized in Table 3.

In addition to their resemblance to natural ECM, the scaffolds can be functionalized with additives, such as ceramics and growth factors, to enhance their biological effects through surface coatings or biomolecule incorporation [63]. Hydroxyapatite, a major component of natural bone, can be incorporated into the fibers to emulate the native inorganic bone component and increase the scaffolds bioactivity and osteoconductivity [64,65]. Through the increase in bioactivity and close mimicry of the native ECM, these nanofibrous scaffolds might have the capacity to recruit host stem and progenitor cells and promote their proliferation and differentiation into fibroblasts, osteoblasts, and cementoblasts, possibly regenerating all the periodontal tissues. Other additives that have been incorporated into the scaffolds include antibiotics [66,67] that confer antibacterial activity, anti-inflammatory drugs [68,69], small molecules [70,71], and gene delivery vectors [72,73], which can enhance the osteogenic and angiogenic properties of the scaffolds.

**Figure 5:**
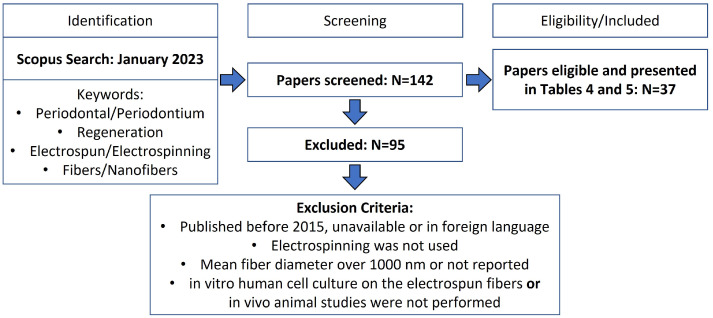
PRISMA flow diagram for the studies retrieved from the search and selection criteria.

The development of electrospun scaffolds for periodontal regeneration envisaging novel GTR membranes or scaffolds to insert in the defect site has been widely researched. Table 4 and Table 5 provide an overview of the recent research studies on electrospun nanofibrous scaffolds along with the specific features and main results from each study. The tables summarize the findings of the literature research carried out as represented in Figure 5. The keywords used were: periodontal regeneration, electrospinning, fibers, and synonyms or alternative words. Papers were excluded if they were published before 2015, did not include in vitro human cell culture on the electrospun scaffolds (for Table 4), or in vivo animal studies were not performed (for Table 5).

Table 4 and Table 5 demonstrate that various strategies have used additives, including growth factors, ceramics, metal oxides, antibiotics, and small molecules. Considering the inflammatory nature of periodontitis, some studies used specific additives to control and reduce inflammation, with clear results from in vitro and in vivo tests [68,69,70]. Because periodontal disease treatment requires removal of bacteria and dental plaque, several studies incorporated metal oxides [78,85,96] or antibiotics, such as metronidazole [66,81,108], tetracycline hydrochloride [67,97,98,99], and amoxicillin [66,104]. The use of metal oxides and antibiotics conferred antibacterial activity to the nanofibrous scaffolds; however, high quantities of the additive often resulted in a decrease in cell viability [81,85,97,98,99,105]. Metal oxides also were shown to enhance the osteogenic effects of the scaffolds, confirmed by increased cell mineralization, upregulated osteogenic gene expression, and elevated ALP activity in vitro [78,79], as well as accelerated new bone formation in vivo [79,109].

The incorporation of osteoinductive or osteoconductive additives, such as bone morphogenetic protein 2, dexamethasone, hydroxyapatite, β-tricalcium phosphate, bioactive glass, and silicate nanoparticles, into nanofibrous scaffolds resulted in augmented osteogenic potential and bioactivity. These scaffolds showed upregulated osteogenic gene expression [71,72,88] and increased cell viability [72,76,103] and promoted bone regeneration in vivo, as reported in various studies [71,88,108,110,111].

Instead of focusing on bone regeneration, one study shifted the focus to cementum regeneration. Chen et al. incorporated recombinant cementum protein 1 (rCMP1) in nanofibrous scaffolds, which downregulated osteogenic gene expression (osteocalcin and osteopontin) and upregulated cementoblastic markers (CMP1 and cementum attachment protein). The scaffolds showed less new bone formation and more cementum-like tissue in rat calvarial defects in vivo [90]. It should be noted that some studies only study bone formation in vivo using calvarial bone defects [75,79,90,110]. It is also relevant to study ligament regeneration, as it plays the essential role of connecting alveolar bone to cementum. For example, rat periodontal defects [69,71,77,106,108,109] or canine periodontal defects [107,112] can be used as more accurate models to study the possible effects of nanofibrous scaffolds in periodontal treatment. Some studies also presented in vivo tests limited to subcutaneous implantation, which were used only to assess the biocompatibility and cell barrier effects of the nanofibrous scaffolds [66,94,95].

The frequent use of natural polymers, such as chitosan, alginate, gelatin, and collagen, to produce nanofibers for periodontal regeneration is evident when analyzing Table 4 and Table 5. Natural polymers enhance the biological effects of the scaffolds, as confirmed by increased cell viability [75,87,89,91], and also improve the scaffolds’ osteogenic potential, as demonstrated by increased cell mineralization [86], ALP activity [91], and new bone formation in vivo [75]. When selecting the polymers to prepare electrospun scaffolds, certain characteristics, such as molecular weight, may significantly impact the scaffolds properties. In one study, low molecular weight chitosan conferred antibacterial activity to the scaffolds, whereas medium molecular weight chitosan did not [101].

Another strategy to improve the bioactivity of scaffolds is the use of proteins to stimulate the differentiation of stem cells. Lam et al. produced core-shell nanofibers loaded with enamel matrix proteins in the core. The use of the enamel matrix derivative Emdogain^®^ resulted in upregulated osteogenic gene expression by PDLSCs. [93] This enamel matrix protein-based gel relies on biological mimicry to stimulate periodontal regeneration. Through the use of native tissue components, such as hydroxyapatite, growth factors, and certain proteins, it is possible to more closely mimic the natural microenvironment, thus facilitating regeneration.

One strategy that has not yet been extensively researched in periodontal regeneration is the use of cell-derived ECM. Cell-derived ECM is a reservoir of proteins and growth factors that influence cell proliferation and differentiation. It consists of secreted ECM by cells cultured in vitro, thus mimicking the composition of native ECM [114,115,116]. Cell-derived ECM has been used in combination with scaffolds in TE, as it mimics the in vivo microenvironment and enhances the scaffolds bioactivity [117,118,119]. Jiang et al. placed PDLSC-sheets on top of PCL/gelatin nanofibers and then decellularized the constructs. Decellularized cell sheets, with and without nanofibers, showed periodontium regeneration potential in rat periodontal defects, confirmed by formation of new bone, cementum, and PDL in vivo [120]. Farag and colleagues transferred PDLSC-sheets onto melt electrospun PCL membranes and then decellularized the constructs [121,122]. Decellularized cell sheets were shown to maintain the ECM intact, retain growth factors, and support recellularization by allogenic PDLSCs in vitro [121]. The decellularized cell-sheets also demonstrated enhanced expression of osteogenic genes by PDLSCs compared to PCL scaffolds alone. Decellularized cell-sheets were biocompatible in vivo and supported periodontal attachment in a rat periodontal defect model [122].

Interestingly, in the majority of the studies, non-aligned nanofibers are produced. From the few studies that fabricated aligned nanofibers, two stand out with in vivo tests. Jiang et al. combined various layers of PCL-PEG nanofibers, either aligned or non-aligned, with a CTS solution and lyophilized the assembly to obtain multilayer scaffolds. Their in vivo performance was evaluated in a rat periodontal defect model. The multilayer scaffolds were placed in contact with the tooth root surface and the defect was filled with the bone graft Bio-Oss^®^ to immobilize the scaffolds. The aligned PCL-PEG nanofibers resulted in a higher expression of periostin, more mature collagen fibers, and significant formation of tooth-supporting mineralized tissue, as well as oriented PDL-like fibers in the regenerated periodontium [106]. Yang et al. produced aligned and non-aligned PCL nanofibers, which were stacked and then immersed in a gelatin solution, creating aligned and non-aligned constructs that were then lyophilized. The aligned construct facilitated collagen formation and maturation, significantly enhanced the angulation of new-born PDL-like tissue, and showed higher periostin expression at periodontal fenestration defects [77]. The regeneration of functional and organized PDL is important, as its unique structure is essential for the physiological functionalities of periodontal tissues.

In addition to the in vivo studies on animals, two clinical trials were recently carried out. Chen et al. studied PLA electrospun microfibrous scaffolds with β-tricalcium phosphate (β-TCP) incorporated in four LanYu pigs and fifteen human periodontal patients. The commercially available PLA dental membrane Epi-guide^®^ was used as a control. PLA/β-TCP scaffolds showed no cytotoxicity in vitro and similar regeneration in vivo compared to the control. The electrospun scaffolds and the control membranes blocked the migration of fast-growing connective tissue into the defect site, created space for new tissue regeneration, and showed increased new cementum and bone formation compared to the blank control. The results from the clinical trials demonstrated significantly more tooth attachment gain, shallower probing depths, and improvement of periodontal inflammation in patients with electrospun scaffolds and the control membranes. In contrast with the patients with electrospun scaffolds, few patients with the control membrane showed soreness at the surgical sites [123]. The study revealed the suitability of electrospun PLA/β-TCP scaffold as an alternative GTR membrane for clinical applications. In another clinical trial, polyvinyl acetate/*Ocimum sanctum* electrospun fibrous scaffolds were placed in patients following scaling and root planing. Patients belonging to the control groups only underwent scaling and root planing without placement of the electrospun scaffolds. Results from this clinical trial showed significant clinical tooth attachment gain in the group treated with electrospun scaffolds. There was significant reduction in the pro-inflammatory cytokine interleukin 1-β(IL-1β) levels between before and after the treatment with scaling and root planing and electrospun scaffold placement. The post-treatment results did not differ significantly from the group undergoing only scaling and root planing [124]. This clinical trial (ID CTRI/2018/07/014961) demonstrated additional benefit when using electrospun fibrous scaffolds together with a non-surgical periodontitis treatment.

## 6. Current Challenges and Future Perspectives

Considering the complexity of the periodontium’s structure and the presence of various cell types, it is important that periodontal treatments promote a coordinated, organized, and hierarchical tissue regeneration. Although many studies report quantitative results related to new alveolar bone formation, this is not necessarily accompanied by PDL and cementum regeneration [125]. The regeneration of functional PDL is a key requirement for periodontal regeneration. However, more accurate periodontal disease models are needed to study PDL formation. Currently available in vitro models cannot recreate the complexity of the PDL, thus limiting a more detailed investigation of the tissue, leading to a dependence on animal studies [126]. More advanced in vitro periodontal disease models are needed and are currently being explored [126].

In order to stimulate a hierarchical tissue regeneration, biomimetic multilayered scaffolds can be produced through the combination of various techniques (e.g., 3D printing, electrospinning, solution casting) [127]. By developing hierarchical scaffolds that emulate the various periodontal tissues, a more synchronized tissue regeneration may occur. Another concern when designing novel biomimetic scaffolds is the spatiotemporal organization of the several cell types present in the periodontium. The PDL provides a cell reservoir necessary to maintain a balance between formation and maintenance of the hard and soft periodontal tissues, therefore scaffolds should support cell migration, proliferation, and differentiation of various cell types. One aspect that should also be taken into consideration is the mechanical stimulation that the PDL is physiologically subjected to. On one hand, advanced in vitro models should mimic the periodontium’s structural organization and reproduce the mechanical loading experienced in vivo. On the other hand, scaffolds should be stimulated mechanically not only to study their features but also to analyze how the stimulation affects cell behavior [128].

Electrospun nanofibrous scaffolds present suitable characteristics to be used as GTR membranes or to be part of multilayered biomimetic constructs for periodontal regeneration, showing promising results both in vitro and in vivo. These features result from a laborious optimization process due to the number of electrospinning parameters to control, which can pose some challenges. Although there are many synthetic and natural polymers, only a number of polymers can satisfy biocompatibility, non-toxicity, degradability, and mechanical properties. The polymers used in the process dictate the basic characteristics of the electrospun scaffolds [36]. It is important to carefully select the most appropriate polymer or combination of polymers to achieve the desired scaffolds features. In addition to polymer selection and dissolution, the electrospinning process and environmental parameters need to be optimized to obtain homogeneous fibers with the desired diameter and alignment and fibrous scaffolds made of cummulative fibers with coveted porosity, mechanical properties, and biodegradability [4]. Additionally, if the electrospinning solution flow rate is slow, the process may take several hours to produce fibrous scaffolds with enough thickness. The morphology of the fibers can be tailored; nevertheless, it may be difficult to control and tune considering the multiple parameters involved, not only from the process but also from the polymeric solution itself. Furthermore, fabricating electrospun fibers with an additive incorporated may result in additional complications, especially when one wishes to control the additive release or homogeneous distribution on the fibers without aggregation [4].

Even though electrospinning may include a laborious optimization process, once all the parameters are set it is crucial to maintain them, with the aim of obtaining reproducible results. For biological and TE applications, it is essential to properly sterilize the scaffolds while avoiding damages to the nanofibers [129]. In the case of scaffolds that require crosslinking, the toxicity of the agent must be taken into consideration and the non-toxicity of the crosslinked scaffolds must be verified. It is important to address the aforementioned limitations and have a good understanding of the scaffolds properties before translating the electrospun scaffolds from the laboratory to clinical applications [129].

This review presented a detailed overview of the use of electrospun nanofibers in periodontal tissue engineering applications, focusing on studies in vitro combining human stem cell cultures with electrospun nanofibers and also in in vivo animal studies, in which the nanofibrous scaffolds were implanted to promote periodontal regeneration. As a result of the chosen exclusion criteria, this review does not include studies with in vitro animal cell culture and papers published before 2015. The exclusion of these papers, which may contain valuable information, represents a limitation of this review.

Taking into account the promising results from the presented in vitro studies and in vivo tests in animal models and the publication of clinical trials using electrospun fibrous scaffolds, more studies on these scaffolds with in vivo tests and possibly more clinical trials will emerge in the near future. However, it is important to note that, although promising results were obtained in vitro with human stem cells and in vivo animal models, the translation into clinical practice is still a difficult concern. Future studies should aim to achieve scaffolds with anti-inflammatory and antibacterial properties, as well as hierarchical regeneration of functional bone-PDL-cementum complexes. The use of appropriate periodontal defect animal models for in vivo testing is necessary, as well as the study of the scaffolds effects on PDL regeneration and new fiber angulation, as it connects alveolar bone to cementum and is vital for a functional periodontium. In the future, the research on nanofibers for periodontal regeneration will continue, either with the development of novel additive-loaded GTR membranes with anti-inflammatory, antibacterial, and bioactive effects, or with the development of multilayer biomimetic scaffolds, in which nanofibers can represent the layer in contact with the root cementum and responsible for cementum and aligned PDL fibers regeneration. Considering its high potential, one strategy that will presumably be further investigated is the use of cell-derived ECM as an additive to provide a closer mimicry of the native tissue composition, thus accelerating tissue regeneration. Additionally, it would be interesting to test nanofibers in combination with other compounds, such as probiotics and other natural-derived molecules. In fact, probiotics have been used in adjunctive treatments to treat periodontitis and demonstrated an influence on plaque and other periodontal parameters [130]. These compounds should be evaluated in combination with nanofibers in future in vivo studies and clinical trials.

## 7. Conclusions

The periodontium is a highly complex structure, consisted of alveolar bone, PDL, and cementum. The PDL is composed of highly organized and aligned collagen fibers, whose organization is essential for the physiology and functionality of the periodontium. Advanced stages of periodontitis lead to inflammation and loss of all periodontal tissues. Possible treatment candidates for periodontal regeneration should promote an hierarchical regeneration of bone-PDL-cementum complexes to restore functional periodontium.

Electrospun nanofibers have high porosity and a high surface area to volume ratio, which, together with the mimicry of the scale and morphology of native ECM, favor cell adhesion, proliferation, and differentiation. Electrospun nanofibers form scaffolds with features suitable for GTR membranes and hierarchical scaffolds. This review provides an overview of the recent studies focused on the use of electrospun nanofibrous scaffolds for periodontal regeneration. These scaffolds can be complemented with various additives to enhance biological characteristics or provide antibacterial, anti-inflammatory, or osteogenic effects. Recent research on electrospun nanofibrous scaffolds shows promising results from in vivo tests, such as enhanced biocompatibility, increased bone formation, formation of newly and well oriented PDL fibers similar to the native tissue, decreased inflammation, and antibacterial activity. However, a biomimetic scaffold able to achieve all these regenerative outcomes is yet to be developed, and the difficult translation from in vitro/in vivo tests into clinical practice must be taken into account.

Future research should focus on the use of appropriate animal models to study the effects of electrospun nanofibrous scaffolds on periodontal regeneration, especially on the formation and alignment of newly formed PDL in addition to bone formation. The production efficiency and reproducibility of these scaffolds still needs to be explored in order to obtain more standardized membranes that can become alternative effective treatment options for periodontitis. The development of highly biomimetic hierarchical scaffolds using electrospun nanofibers and cell-derived ECM to facilitate periodontal regeneration holds great promise for the near future.

## Figures and Tables

**Table 1:** Currently available non-surgical and surgical treatments for periodontal disease.

Non-Surgical	Surgical
Plaque control [26]	Open flap surgery [27]
Scaling and root planing [28]	Pocket reduction [29]
Adjunctive therapies [30]	Guided tissue regeneration [31]
Laser treatment [32]	Bone grafting [33]

**Table 2:** Sources of stem cells for periodontal regeneration.

Dental Tissues			Other Tissues
Periodontal Ligament [51]	Dental Pulp [53]	Gingiva [50]	Bone Marrow [49]
Exfoliated Deciduous Teeth [54]	Dental Follicle [55]		Adipose Tissue [56]

**Table 3:** Physical, mechanical, and chemical properties of electrospun nanofibers.

Physical	Mechanical	Chemical
Fiber diameter	Ultimate tensile stress	Composition Hydrophilicity
Fiber alignment	Elastic modulus Elongation	Functionalization (e.g., additives)

**Table 4:** Examples of in vitro studies using electrospun nanofibers seeded with human cells for periodontal regeneration.

Polymers + Additives	MFD (nm)	Alignment	Cells	Main Outcomes from In Vitro Culture Studies	Year; Ref
CTS, PEG	410 ± 163 288 ± 107	aligned non-aligned	ES-MPs	Non-aligned fibers: ↑ calcium deposition by ES-MPs; Aligned fibers: ↑ cell viability	2017; [74]
COL, CTS, PCL	239 ± 26	non-aligned	PDLCs	Membranes with CTS → ↑ cell viability	2020; [75]
GEL, PEG + HAP	528 ± 17	non-aligned	BMMSCs PDLCs	↑ porosity → ↑ cell viability; ↑ POSTN and OPN expression in cocultures	2022; [76]
PCL, GEL	599 ± 95 590 ± 167	aligned non-aligned	PDLSCs	Both types non-cytotoxic; cells elongated along fiber alignment on aligned fibers	2019; [77]
PCL//GEL + MgO	≈400	non-aligned	PDLSCs	MgO NPs in the core → antibacterial + ↑ mineralization + ↑ ALP and RUNX2	2021; [78]
PCL, GEL + CeO_2_	378 ± 204 355 ± 181	non-aligned	PDLSCs	↑ cell proliferation and ALP activity in membranes with CeO_2_ NPs	2022; [79]
PCL, PEG	522 ± 159	non-aligned	PDLSCs	↑ ALP, RUNX2, OC gene expression	2021; [80]
PU + MET	200–300	non-aligned	DPSCs	↑ metronidazole content→ ↓ cell viability	2020; [81]
PLGA + pFGF-2	165 ± 60	non-aligned	PDLCs	pFGF-2 → ↑ cell viability; ↑ COL I and scleraxis gene expression	2020; [73]
SF, PEG	300–400	non-aligned	PDLCs	Sonication+↓ PEG→ ↑ cell proliferation	2019; [82]
PLGA, WK + ORN or bFGF	600–700	non-aligned	PDLCs	ORN or bFGF→ ↑ proliferation; ↑ ALP; cytocompatible but ↓ with ↑ ORN/bFGF	2020; [83] [84]
PCL, GEL + ZnO	250–650	non-aligned	DPSCs	Antibacterial due to ZnO NPs; cytocompatible (but ↓ with ↑ ZnO)	2015; [85]
PLA, CTS	≈200	non-aligned	PDLCs; BMMSCs	↑ pro-inflammatory mediators expression by PDLCs; ↑ CTS NPs → ↑ hydrophilic, ↑ OPG and mineralization by BMMSCs	2018; [86]
PLA, Calcium alginate	250 ± 90	non-aligned	PDLCs; BMMSCs	↑ COL I, RUNX2 and OPG by BMMSCs and ↑ pro-inflammatory levels by PDLCs; Alginate → ↑ hydrophilic, ↑ cell viability	2020; [87]
COL, CTS + BG	159 ± 59	non-aligned	PDLCs	↑ cell viability; ↑ RUNX2, OPN, OC, ALP gene expression; antibacterial activity	2017; [88]
SF, PEG + vancomycin	≈500	non-aligned	PDLCs	Antibacterial due to vancomycin loaded GEL NSs; ↑ GEL NSs → ↑ cell proliferation	2017; [89]
PCL + DOX	150–300	non-aligned	PDLCs	↑ DOX → antibacterial, but ↓ cell viability	2016; [16]
PCL, COL, PEG + rCMP1	≈200	non-aligned	PDLCs	rCMP1 → ↑ CMP1, CAP; ↓ OC, OPN	2016; [90]
Zein, GEL	407 ± 140	non-aligned	PDLSCs	GEL → ↑ cell proliferation; ↑ ALP activity	2017; [91]
PCL	377 ± 3	non-aligned	DFSCs	Fibroblastic differentiation	2016; [92]
PLGA + DMOG, nSi	1069 922	non-aligned	PDLSCs	DMOG→ ↑ VEGF; nSi→ ↑ ALP, OC, OPN RUNX2; induced angio- and osteogenesis	2021; [71]
PCL//PEG + EMD	500–1000	non-aligned	PDLSCs	Enamel Matrix Derivative in the core → ↑ OC, RUNX2, ALP and OPN expression	2021; [93]
PCL, GEL	500–600	non-aligned	GFs	↑ cell viability; ↑ COL secretion/deposition	2020; [94]
PLGA, GEL	322 ± 58	aligned	DFSCs	Cell proliferation along fiber direction; ↓ ALP, RUNX2; ↑ VEGF gene expression	2015; [95]
PLGA or PCL + MET, AMX	240 ± 48 282 ± 68	non-aligned	GFs	Both: > 80 % cell viability with different drug concentrations; PCL → ↑ cell viability	2021; [66]
PLA + CaO_2_, MnO_2_	≈300	non-aligned	NOK	Sustained oxygen release; 7.5% CaO_2_ + MnO_2_ → antibacterial and ↑ cell viability	2022; [96]
PCL, Zein + β-GP//PEG + Curc, TH	150–300	non-aligned	NOK	Coaxial fibers on the surface of 3D-printed layer; TH → antibacterial; Biocompatible but ↑ Curc/TH release → ↓ cell viability	2022; [97]
PLCL + TH	≈600	non-aligned	OK; OF	OF > OK viability; ↑ TH → ↓ cell viability	2022; [98]
CTS//PVA + TH	150–300	non-aligned	DF	Cytocompatible; TH → antibacterial	2020; [67]
PLGA//GT + TH	200–400	non-aligned	DF	Coaxial → ↑ prolonged TH release than blend; cytocompatible; TH → ↓ cell viability	2016; [99]
PCL	300–800	non-aligned	DF	Biocompatible; cell barrier properties	2019; [100]
GEL, CTS + CA	350–500	non-aligned	DF SAOS-2	Biocompatible; only scaffolds with low molecular weight CTS → antibacterial	2021; [101]
GEL + SP600125, SB203580	≈350	non-aligned	PDLCs	Cytocompatible: similar cell proliferation on loaded fibers, pure GEL fibers and TCP; ↓ MMP-2 and MMP-13 expression	2019; [102]
PVA + BR, Mg-HAP	200 ± 15 300 ± 14	non-aligned	GF	Surface of a porous scaffold; BR/Mg-HAP →↑ cell adhesion, proliferation, migration	2020; [103]
PLA + AMX	737 ± 128 775 ± 174	non-aligned	PDLCs	Cell viability similar to TCP; ↑ cyclin D and ROCK II expression; AMX → antibacterial	2021; [104]
PLGA//PEI + pBMP-2	300–500	non-aligned	PDLSCs	Blend and coaxial show similar cell viability; Coaxial → ↑ BMP-2, RUNX2 expression, ↑ calcium levels, ↑ transfection efficiency	2016; [72]
PLA + QUE	≈500	non-aligned	GF	QUE release in acidic conditions; QUE → ↓ pro-inflammatory mediator expression	2022; [68]
CTS, PEG + Ag, HAP, Si, DOXH	150–200	non-aligned	GF	Biocompatible; DOXH → ↓ cell viability; antibacterial due to DOXH and Ag/HAP/Si	2022; [105]

**Table 5:** Examples of in vivo studies on electrospun nanofibers for periodontal regeneration.

Polymers + Additives	MFD (nm)	Alignment	Model	Main Outcomes from In Vivo Studies	Year; Ref.
PCL, COL, PEG + rCMP1	≈200	non-aligned	rat	rCMP1 → ↑ cementum-like tissue and ↓ new bone formation in defect	2016; [90]
COL, CTS, PCL	239 ± 26	non-aligned	rat	Membranes with CTS → ↑ new bone formed; ↑ bone ALP and OC expression	2020; [75]
PCL, GEL	599 ± 95 590 ± 167	aligned non-aligned	rat	Aligned Fibers → ↑ new oriented PDL fibers similar to natural PDL, ↑ POSTN	2019; [77]
COL, CTS + BG	159 ± 59	non-aligned	dog	↑ new bone formation; ↓ inflammation compared to the control (no membrane)	2017; [88]
PCL, GEL + CeO_2_	378 ± 204 355 ± 181	non-aligned	rat	Membranes with CeO_2_ NPs accelerated new bone formation	2022; [79]
PLGA + pFGF-2	165 ± 60	non-aligned	dog	pFGF-2 → ↓ root surface resorption; more regular PDL-like tissues formed	2020; [73]
PLGA + DMOG, nSi	1069 922	non-aligned	rat	DMOG/nSi→ functional cementum- PDL-bone complex; ↑ new bone formed	2021; [71]
PCL, PEG	616 ± 213 574 ± 218	aligned non-aligned	rat	Aligned fibers: ↑ new PDL-like oriented fibers; ↑ COL I, ↓ COL III; ↑ POSTN	2015; [106]
PCL, GEL	500–600	non-aligned	rat	Biodegradable; strong cell barrier effects	2020; [94]
PLGA, GEL	322 ± 58	aligned	swine	Layer of a p-DFSCs seeded construct → cementum and PDL-like tissues formed	2015; [95]
PLGA + MWNTs	<1000	non-aligned	dog	↑ new cementum, PDL and bone formed compared to the control (no membrane)	2018; [107]
PVA + HAP, MET	200–300	non-aligned	rat	MET → antibacterial, disease control; HAP → significant ↓ in pocket depth	2018; [108]
PLA + MgO	≈500	non-aligned	rat	MgO NPs → ↑ new bone formation	2020; [109]
PCL, GEL + BG, DEX	<1000	non-aligned	rat	Biocompatible with and without DEX; DEX→ ↑ bone volume and surface density	2015; [110]
GEL + β-TCP	≈400	non-aligned	rabbit	β-TCP → ↑ bone volume; ↑ OC	2015; [111]
PLGA, WK	200–500	non-aligned	dog	Results similar to COL membrane: ↑ new cementum, PDL and bone; ↑ bone density than with periodontal flap surgery	2016; [112]
PCL, PVP + ZIF-8, FK506	797 ± 138 654 ± 332	non-aligned	rat	ZIF-8/FK506 → ↑ bone volume; ↑ OC; antibacterial + anti-inflammatory effects	2022; [69]
PLGA, GEL + TP, APR	200–400	non-aligned	rat	TP/APR → ↑ bone volume; ↑ dense PDL with ↑ COL fibers; anti-inflammatory	2022; [70]
COL	50–300	non-aligned	rabbit	Layer of COL nanofibers on CTS film → ↑ new bone formed (similar to Bio-Gide)	2016; [113]
PLGA or PCL + MET, AMX	240 ± 48 282 ± 68	non-aligned	rat rabbit	Prolonged drug release; Biocompatible; ↓ inflammation in rabbits than in rats	2021; [66]
GEL + rBMP-2, SP600125, SB203580	≈350	non-aligned	dog	Both inhibitors → ↑ bone volume; larger angulation of the regenerated PDL fibers, closer to natural PDL	2019; [102]
PVA + BR, Mg-HAP	200 ± 15 300 ± 14	non-aligned	rat	BR/Mg-HAP → ↑ oral wound healing rate; more regular arrangement of COL fibers; BR → antibacterial agent	2020; [103]
PLA + AMX	737 ± 128 775 ± 174	non-aligned	rat	AMX → ↑ dense-packed and well aligned regenerated COL fibers; ↓ inflammation	2021; [104]

Abbreviations in Table 4 and Table 5 in order of appearance: **Polymers**: CTS: chitosan, PEG: polyethylene glycol, COL: collagen, PCL: polycaprolactone, GEL: gelatin, shell//core: coaxial fibers, PU: polyurethane, PLGA: poly(lacticco- glycolic acid), SF: silk fibroin, WK: wool keratin, PLA: polylactic acid, PLCL: Poly(l-lactide-co-ε-caprolactone), PVA: polyvinyl alcohol, GT: gum tragacanth, PEI: polyethylenimine, PVP: Polyvinylpyrrolidone; **Additives**: HAP: hydroxyapatite, MgO: magnesium oxide, CeO_2_: cerium oxide, pFGF-2: plasmid DNA encoding fibroblast growth factor-2, ORN: ornidazole, bFGF: basic fibroblast growth factor, ZnO: zinc oxide, BG: bioactive glass, DOX: doxycycline hydrochloride, rCMP1: recombinant cementum protein 1, DMOG: dimethyloxalylglycine, nSi: nanosilicate, EMD: enamel matrix derivative, MET: metronidazole, AMX: amoxicillin, CaO_2_: calcium peroxide, MnO_2_: manganese dioxide, β-GP: β-glycerophosphate disodium salt hydrate, Curc: curcumin, TH: tetracycline hydrochloride, CA: citric acid, SP600125: a JNK inhibitor, SB203580: a p38 inhibitor, BR: bromelain, pBMP-2: Bone Morphogenic Protein 2 plasmid, QUE: quercetin, Ag: silver, Si: silica, DOXH: doxycycline hyclate, MWNTs: multi-walled carbon nanotubes, DEX: dexamethasone, β-TCP: β-tricalcium phosphate, ZIF-8: zeolitic imidazolate framework-8, FK506: tacrolimus, TP: tea polyphenols, APR: adipoRon; Cells: ES-MPs: embryonic stem cellderived mesenchymal progenitor cells, PDL(S)Cs: PDL (stem) cells, BMMSCs: bone marrow-derived MSCs, GF: gingival fibroblasts, (N)OK: (normal) oral keratinocytes, OF: oral fibroblasts, DF: dermal fibroblasts, SAOS-2: a human primary osteogenic sarcoma cell line; **Results**: POSTN: periostin, OPN: osteopontin, NPs: nanoparticles, ALP: alkaline phosphatase, RUNX2: runt-related transcription factor 2, OC: osteocalcin, OPG: osteoprotegerin, NSs: nanospheres, CAP: cementum attachment protein, VEGF: vascular endothelial growth factor, TCP: tissue culture polystyrene, MMPs: matrix metalloproteinases, ROCK II: Rho-associated protein kinase II.

## Data Availability

Not applicable.
